# Adeninium cytosinium sulfate

**DOI:** 10.1107/S1600536809034023

**Published:** 2009-08-29

**Authors:** Aouatef Cherouana, Raja Bousboua, Lamia Bendjeddou, Slimane Dahaoui, Claude Lecomte

**Affiliations:** a Laboratoire de Chimie Moléculaire, du Contrôle de l’Environnement et des Mesures Physico-Chimiques, Faculté des Sciences, Département de Chimie, Université Mentouri de Constantine, 25000 Constantine, Algeria; b Cristallographie, Résonance Magnétique et Modélisation (CRM2), Université Henri Poincaré, Nancy 1, Faculté des Sciences, BP 70239, 54506 Vandoeuvre lès Nancy CEDEX, France

## Abstract

In the title compound, C_5_H_6_N_5_^+^·C_4_H_6_N_3_O^+^·SO_4_^2−^, the adeninium (AdH^+^) and cytosinium (CytH^+^) cations and sulfate dianion are involved in a three-dimensional hydrogen-bonding network with four different modes, *viz.* AdH^+^⋯AdH^+^, AdH^+^⋯CytH^+^, AdH^+^⋯SO_4_^2−^ and CytH^+^⋯SO_4_^2−^. The adeninium cations form N—H⋯N dimers through the Hoogsteen faces, generating a characteristic *R*_2_^2^(10) motif. This AdH^+^⋯AdH^+^ hydrogen bond in combination with AdH^+^⋯CytH^+ ^H-bonds leads to two-dimensional cationic ribbons parallel to the *a* axis. The sulfate anions inter­link the ribbons into a three-dimensional hydrogen-bonding network and thus reinforce the crystal structure.

## Related literature

Nucleobases possess multiple hydrogen-bonding sites (Saenger, 1984 ▶) and so can form an abundance of aggregates through hydrogen bonds, from dimers to infinite extended species, see: Jai-nhuknan *et al.* (1997 ▶); Bendjeddou *et al.* (2003 ▶); Smith *et al.* (2005 ▶); Sridhar & Ravikumar (2007 ▶). For protonated nucleobases in acid-base catalysis, see: Lippert (2005 ▶). For their use in the construction of highly ordered supra­molecular nanostructures which are of inter­est for their potential applications as mol­ecular devices, see: Lehn (1995 ▶); Gottarelli *et al.* (2000 ▶). Bond lengths in adeninium cations are dependent on the degree of protonation, see: Hingerty *et al.* (1981 ▶); Langer & Huml (1978 ▶). For bond angles in neutral adenine, see: Voet & Rich (1970 ▶). For related structures with a cytosinium cation, see: Prabakaran *et al.* (2001 ▶); Smith *et al.* (2005 ▶); Sridhar & Ravikumar (2008 ▶). For graph-set motifs, see: Bernstein *et al.* (1995 ▶). For hydrogen bond ing, see: Jeffrey & Saenger (1991 ▶). For pKa values for cytosine, see: Stecher (1968 ▶).
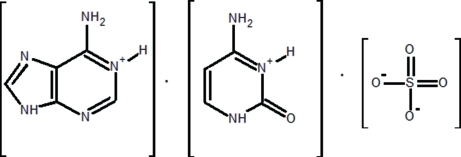


## Experimental

### 

#### Crystal data

C_5_H_6_N_5_^+^·C_4_H_6_N_3_O^+^·SO_4_^2−^
*M* *_r_* = 344.33
Monoclinic, 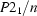

*a* = 9.180 (2) Å
*b* = 12.948 (3) Å
*c* = 11.328 (3) Å
β = 99.356 (2)°
*V* = 1328.6 (5) Å^3^
*Z* = 4
Mo *K*α radiation
μ = 0.29 mm^−1^
*T* = 100 K
0.39 × 0.26 × 0.12 mm

#### Data collection

Oxford Diffraction Xcalibur Saphire2 CCD diffractometer
Absorption correction: analytical (*CrysAlis RED*; Oxford Diffraction, 2008 ▶) *T*_min_ = 0.921, *T*_max_ = 0.975
57856 measured reflections
5843 independent reflections
5061 reflections with *I* > 2σ(*I*)
*R*_int_ = 0.026

#### Refinement

*R*[*F* ^2^ > 2σ(*F* ^2^)] = 0.027
*wR*(*F* ^2^) = 0.088
*S* = 1.04
5843 reflections
232 parameters
8 restraints
H atoms treated by a mixture of independent and constrained refinement
Δρ_max_ = 0.51 e Å^−3^
Δρ_min_ = −0.51 e Å^−3^

### 

Data collection: *CrysAlis CCD* (Oxford Diffraction, 2008 ▶); cell refinement: *CrysAlis RED* (Oxford Diffraction, 2008 ▶); data reduction: *CrysAlis RED*; program(s) used to solve structure: *SIR92* (Altomare *et al.*, 1993 ▶); program(s) used to refine structure: *SHELXL97* (Sheldrick, 2008 ▶); molecular graphics: *ORTEP-3* (Farrugia, 1997 ▶) and *Mercury* (Macrae *et al.*, 2006 ▶); software used to prepare material for publication: *PLATON* (Spek, 2009 ▶).

## Supplementary Material

Crystal structure: contains datablocks global, I. DOI: 10.1107/S1600536809034023/at2867sup1.cif

Structure factors: contains datablocks I. DOI: 10.1107/S1600536809034023/at2867Isup2.hkl

Additional supplementary materials:  crystallographic information; 3D view; checkCIF report

## Figures and Tables

**Table 1:** Hydrogen-bond geometry (Å, °)

*D*—H⋯*A*	*D*—H	H⋯*A*	*D*⋯*A*	*D*—H⋯*A*
N1*A*—H1*A*⋯O1^i^	0.877 (10)	2.535 (10)	3.1036 (19)	123.3 (8)
N1*A*—H1*A*⋯O4^i^	0.877 (10)	1.928 (11)	2.7833 (17)	164.5 (11)
N1*C*—H1*C*⋯O3	0.864 (9)	1.877 (10)	2.7350 (17)	172.0 (11)
N2*C*—H2*C*⋯O1^ii^	0.864 (11)	1.902 (11)	2.7596 (17)	171.8 (11)
N2*A*—H3*A*⋯N7*A*^iii^	0.873 (9)	2.081 (10)	2.9118 (18)	158.7 (12)
N3*C*—H3*C*⋯O4^iv^	0.882 (8)	1.835 (8)	2.7164 (17)	178.2 (11)
N2*A*—H4*A*⋯O5*C*^v^	0.858 (10)	2.102 (10)	2.8368 (18)	143.3 (9)
N2*C*—H4*C*⋯O2^iv^	0.864 (8)	1.901 (8)	2.7622 (17)	174.4 (11)
N9*A*—H9*A*⋯O3^vi^	0.872 (8)	1.870 (8)	2.7364 (17)	172.5 (12)
C2*A*—H2*A*⋯O1^i^	0.9300	2.3900	3.0553 (19)	128.00
C5*C*—H5*C*⋯O2^ii^	0.9300	2.4600	3.357 (2)	161.00
C6*C*—H6*C*⋯N3*A*^v^	0.9300	2.5300	3.447 (2)	170.00
C8*A*—H8*A*⋯O5*C*^iv^	0.9300	2.4200	3.245 (2)	148.00

Symmetry codes: (i) 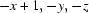; (ii) 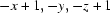; (iii) 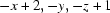; (iv) 
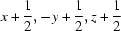
; (v) 
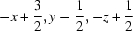
; (vi) 
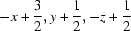
.

**Fig. 1.**
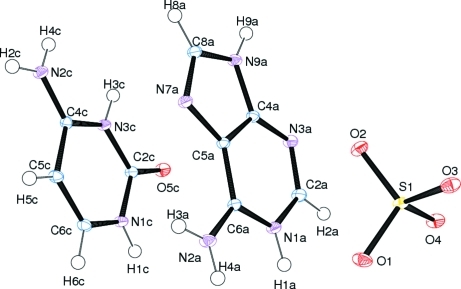
The title molecule with the atom-numbering scheme. The displacement ellipsoids are drawn at the 50% probability level

**Fig. 2.**
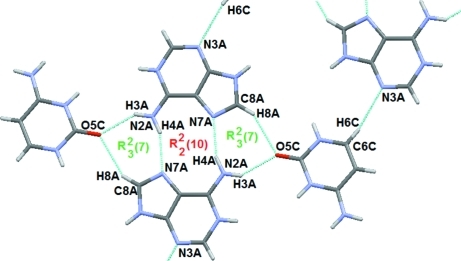
The cation-cation (AdH+···AdH+ and AdH+···CytH+) hydrogen bonds.

**Fig. 3.**
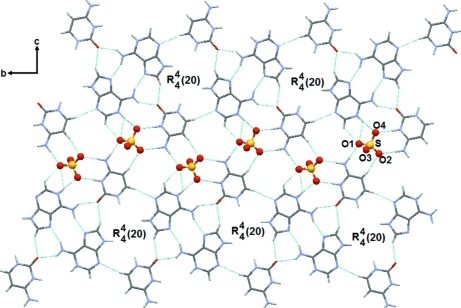
Hydrogen bonding cationic two-dimensional ribbons. The axis a is directed downwards from the projection plane.

## supplementary crystallographic information

### Comment

Nucleobases can be protonated and thus form various cations. They possess multi-hydrogen-bonding sites and various tautomers (Saenger, 1984), such that they can form an abundance of aggregates through hydrogen bonds, from dimers to infinite extended species (Jai-nhuknan *et al.*, 1997; Bendjeddou *et al.*, 2003; Smith *et al.*, 2005; Sridhar & Ravikumar, 2007).

The protonated nucleobases are present in many biochemical processes, such as enzymatic reactions and the stabilization of triplex structures, and they play a key role in a newly emerging feature of nucleic acid chemistry, namely acid-base catalysis (Lippert, 2005). There ability to form hydrogen-bonded networks is obviously the most important and interesting characteristic, because the self-assembly of hydrogen-bonded networks of these compounds or there derivatives has been used to design or construct highly ordered supramolecular nanostructures which are of interest for their potential applications as molecular devices (Lehn, 1995 & Gottarelli *et al.*, 2000).

The main purpose of the present study is to examine the hydrogen bonding engineered in crystal formed by two monoprotonated nucleobases and one dianion: adeninium cytosinium sulfate [AdH^+^, CytH^+^, SO_4_^2-^].

Adeninium cations can be either mono- or diprotonated and the bond lengths and angles are dependent on the degree of protonation (Hingerty *et al.*, 1981; Langer & Huml, 1978). This form contains three basic N atoms, the most basic site is N1, which accepts the first proton, and the next protonation occurs at N7 and then at N3.

The adeninium cation in this structure is monoprotonated at N1 atom. The protonation on this site is evident from the C—N—C bond angle, indeed we note an increase in the C2A—N1A—C6A bond angle [123.35 (6)°] compared with the corresponding value found in the neutral adenine [119.8°; Voet & Rich, 1970]. The location of the H-atom bonded to N1 in a difference Fourrier map and the successful refinement of its position confirms the protonation on this site.

Cytosine is quite a strong base (pKa1 = 1. 6 and pKa2 = 12.2; Stecher, 1968) and, in the presence of acids, is readily protonated at the N3 ring position. The N3 protonation of the cytosine ring in [AdH^+^, CytH^+^, SO_4_^2-^] is consistent with the larger C2C—N3C—C4C angle [124.30 (6)], and with the location of this H-atom in a difference Fourrier map with the successful refinement of its position. The molecular geometries of the cytosinium cation are in good agreement with those of similar structures (Prabakaran *et al.*, 2001; Sridhar & Ravikumar, 2008; Smith *et al.*, 2005).

In the sulfate anion, S atom is linked to four equivalents short bonds of 1.4706 (10) Å to O1 and O2, 1.4895 (10) Å to O3 and 1.4905 (10) Å to O4, which confirm the absence of proton in this anion.

The asymetric unit, of the title compound, is thus formed by one adeninium cation, one cytosinium cation and one sulfate dianion (Fig. 1).

The three-dimensional crystal structure is stabilized by thirteen hydrogen bonds with four different modes *viz.* AdH^+^···AdH^+^, AdH^+^···CytH^+^, AdH^+^···SO_4_^2-^ and CytH^+^···SO_4_^2- ^(Table 1).

The alone AdH^+^···AdH^+ ^hydrogen bond involving the Hoogsteen faces (atoms N2A and N7A) of the adeninium cation form a centrosymmetric dimer generating a characteristic *R_2_^2^(10) *motif (Bernstein *et al.*, 1995) (Fig. 2).

Cytosinium cation is linked to adeninium throught three hydrogen bonds where O5C and C6C acts as acceptor and donor respectively (Table 1). The oxygen atom O5C is involving with two symmetric adeninium cations into a three-centred hydrogen-bonding pattern (Jeffrey & Saenger, 1991). The combination of this three-centred hydrogen bond with N2A—H3A···N7A (AdH^+^···AdH^+^) generates a ring with *R_3_^2^(7)* motif (Fig. 2). The weak C6C—H6C···N3A forms with C8A—H8A···O5C a *R_4_^4^(20)* ring and interlink cations into a two-dimentional ribbons developping along *a* axis (Fig. 3).

AdH^+^···SO_4_^2- ^and CytH^+^···SO_4_^2- ^hydrogen bonds ensure junction between the cationic ribbons into a three-dimensional hydrogen bonding network.

### Experimental

Colourless needle crystals of the title compound [AdH^+^, CytH^+^, SO_4_^2-^], were obtained by slow evaporation at room temperature of an equimolar solution of adenine, cytosine and sulfuric acid.

### Refinement

All the H atoms were located in the difference electron density maps. All the H atoms attached to C were treated as riding with C—H = 0.93 Å (aromatic) with *U*_iso_(H) = 1.2*U*_eq_(C). The coordinate parameters of the H atoms attached to N were freely refined with *U*_iso_(H) = 1.2*U*_eq_(N).

### Crystal data

**Table:**

C_5_H_6_N_5_^+^·C_4_H_6_N_3_O^+^·SO_4_^2^^−^	*F*(000) = 712
*M**_r_* = 344.33	*D*_x_ = 1.721 Mg m^−^^3^
Monoclinic, *P*2_1_/*n*	Mo *K*α radiation, λ = 0.71073 Å
Hall symbol: -P 2yn	Cell parameters from 57856 reflections
*a* = 9.180 (2) Å	θ = 3.1–35.0°
*b* = 12.948 (3) Å	µ = 0.29 mm^−^^1^
*c* = 11.328 (3) Å	*T* = 100 K
β = 99.356 (2)°	Prism, colourless
*V* = 1328.6 (5) Å^3^	0.39 × 0.26 × 0.12 mm
*Z* = 4	

### Data collection

**Table:**

Oxford Diffraction Xcalibur Saphire2 CCD diffractometer	5843 independent reflections
Radiation source: fine-focus sealed tube	5061 reflections with *I* > 2σ(*I*)
graphite	*R*_int_ = 0.026
φ and ω scans	θ_max_ = 35.0°, θ_min_ = 3.1°
Absorption correction: analytical (*CrysAlis RED*; Oxford Diffraction, 2008)	*h* = −14→14
*T*_min_ = 0.921, *T*_max_ = 0.975	*k* = −20→20
57856 measured reflections	*l* = −17→18

### Refinement

**Table:**

Refinement on *F*^2^	Primary atom site location: structure-invariant direct methods
Least-squares matrix: full	Secondary atom site location: difference Fourier map
*R*[*F*^2^ > 2σ(*F*^2^)] = 0.027	Hydrogen site location: difference Fourier map
*wR*(*F*^2^) = 0.088	H atoms treated by a mixture of independent and constrained refinement
*S* = 1.04	*w* = 1/[σ^2^(*F*_o_^2^) + (0.0602*P*)^2^ + 0.1819*P*] where *P* = (*F*_o_^2^ + 2*F*_c_^2^)/3
5843 reflections	(Δ/σ)_max_ = 0.001
232 parameters	Δρ_max_ = 0.51 e Å^−^^3^
8 restraints	Δρ_min_ = −0.51 e Å^−^^3^

### Special details

**Table:**

Experimental. CrysAlis RED (Oxford Diffraction, 2008) Analytical numeric absorption correction using a multifaceted crystal model based on expressions derived by R.C. Clark & J.S. Reid.
Geometry. Bond distances, angles *etc*. have been calculated using the rounded fractional coordinates. All su's are estimated from the variances of the (full) variance-covariance matrix. The cell e.s.d.'s are taken into account in the estimation of distances, angles and torsion angles
Refinement. Refinement of *F*^2^ against ALL reflections. The weighted *R*-factor *wR* and goodness of fit *S* are based on *F*^2^, conventional *R*-factors *R* are based on *F*, with *F* set to zero for negative *F*^2^. The threshold expression of *F*^2^ > σ(*F*^2^) is used only for calculating *R*-factors(gt) *etc*. and is not relevant to the choice of reflections for refinement. *R*-factors based on *F*^2^ are statistically about twice as large as those based on *F*, and *R*- factors based on ALL data will be even larger.

### Fractional atomic coordinates and isotropic or equivalent isotropic displacement parameters (Å^2^)

**Table:**

	*x*	*y*	*z*	*U*_iso_*/*U*_eq_	
N1A	0.89503 (7)	0.08114 (5)	0.15030 (6)	0.0102 (1)	
N2A	0.93054 (8)	−0.04224 (5)	0.30191 (6)	0.0114 (2)	
N3A	0.92695 (7)	0.26232 (5)	0.17622 (6)	0.0113 (2)	
N7A	1.01108 (7)	0.14551 (5)	0.46688 (6)	0.0097 (1)	
N9A	1.00189 (7)	0.30483 (5)	0.38697 (6)	0.0106 (2)	
C2A	0.89521 (8)	0.18034 (6)	0.10957 (7)	0.0113 (2)	
C4A	0.96300 (8)	0.23844 (5)	0.29397 (6)	0.0091 (2)	
C5A	0.96826 (7)	0.14027 (5)	0.34460 (6)	0.0083 (2)	
C6A	0.93243 (7)	0.05505 (5)	0.26787 (6)	0.0087 (1)	
C8A	1.02951 (8)	0.24570 (5)	0.48772 (7)	0.0106 (2)	
O5C	0.58498 (6)	0.24982 (4)	0.27802 (5)	0.0133 (2)	
N1C	0.59982 (7)	0.08175 (5)	0.33878 (6)	0.0100 (1)	
N2C	0.76585 (7)	0.18374 (5)	0.67114 (6)	0.0107 (2)	
N3C	0.67261 (7)	0.21469 (5)	0.47342 (6)	0.0092 (1)	
C2C	0.61681 (8)	0.18570 (5)	0.35794 (7)	0.0093 (2)	
C4C	0.71397 (7)	0.14707 (5)	0.56532 (7)	0.0085 (2)	
C5C	0.69956 (8)	0.03932 (5)	0.54009 (7)	0.0100 (2)	
C6C	0.64100 (8)	0.01083 (5)	0.42725 (7)	0.0102 (2)	
S1	0.27538 (2)	0.03329 (1)	0.11795 (2)	0.0083 (1)	
O1	0.19268 (7)	−0.06314 (4)	0.12530 (5)	0.0137 (2)	
O2	0.24941 (6)	0.10652 (4)	0.21156 (5)	0.0119 (1)	
O3	0.43624 (6)	0.01038 (4)	0.13141 (5)	0.0122 (1)	
O4	0.22552 (6)	0.07936 (4)	−0.00242 (5)	0.0115 (1)	
H1A	0.8721 (13)	0.0315 (8)	0.0979 (9)	0.0123*	
H2A	0.87069	0.19049	0.02747	0.0135*	
H3A	0.9548 (13)	−0.0569 (10)	0.3778 (8)	0.0136*	
H4A	0.9167 (13)	−0.0894 (8)	0.2481 (9)	0.0136*	
H8A	1.05853	0.27310	0.56380	0.0127*	
H9A	1.0154 (13)	0.3714 (6)	0.3849 (11)	0.0127*	
H1C	0.5516 (12)	0.0640 (9)	0.2698 (8)	0.0120*	
H2C	0.7866 (13)	0.1437 (9)	0.7326 (9)	0.0129*	
H3C	0.6899 (12)	0.2814 (6)	0.4829 (10)	0.0111*	
H4C	0.7671 (12)	0.2495 (6)	0.6843 (10)	0.0129*	
H5C	0.72934	−0.00972	0.59907	0.0120*	
H6C	0.62847	−0.05905	0.40948	0.0122*	

### Atomic displacement parameters (Å^2^)

**Table:**

	*U*^11^	*U*^22^	*U*^33^	*U*^12^	*U*^13^	*U*^23^
N1A	0.0133 (2)	0.0090 (2)	0.0078 (3)	−0.0006 (2)	−0.0001 (2)	−0.0007 (2)
N2A	0.0175 (3)	0.0066 (2)	0.0096 (3)	−0.0002 (2)	0.0010 (2)	−0.0009 (2)
N3A	0.0146 (3)	0.0097 (2)	0.0088 (3)	−0.0012 (2)	−0.0001 (2)	0.0010 (2)
N7A	0.0122 (2)	0.0085 (2)	0.0080 (3)	−0.0007 (2)	0.0007 (2)	−0.0006 (2)
N9A	0.0145 (3)	0.0070 (2)	0.0098 (3)	−0.0019 (2)	0.0008 (2)	−0.0005 (2)
C2A	0.0141 (3)	0.0104 (3)	0.0087 (3)	−0.0007 (2)	−0.0002 (2)	0.0012 (2)
C4A	0.0107 (3)	0.0076 (3)	0.0089 (3)	−0.0010 (2)	0.0009 (2)	−0.0001 (2)
C5A	0.0101 (3)	0.0070 (2)	0.0077 (3)	−0.0005 (2)	0.0010 (2)	−0.0002 (2)
C6A	0.0097 (2)	0.0080 (2)	0.0082 (3)	0.0002 (2)	0.0010 (2)	−0.0002 (2)
C8A	0.0133 (3)	0.0093 (3)	0.0089 (3)	−0.0015 (2)	0.0006 (2)	−0.0006 (2)
O5C	0.0198 (3)	0.0095 (2)	0.0093 (3)	0.0003 (2)	−0.0017 (2)	0.0021 (2)
N1C	0.0126 (2)	0.0077 (2)	0.0088 (3)	−0.0007 (2)	−0.0013 (2)	−0.0008 (2)
N2C	0.0144 (3)	0.0091 (2)	0.0079 (3)	−0.0005 (2)	−0.0006 (2)	0.0002 (2)
N3C	0.0127 (2)	0.0064 (2)	0.0078 (3)	−0.0001 (2)	−0.0007 (2)	0.0001 (2)
C2C	0.0103 (3)	0.0081 (3)	0.0089 (3)	−0.0001 (2)	0.0002 (2)	−0.0002 (2)
C4C	0.0089 (2)	0.0082 (3)	0.0084 (3)	0.0000 (2)	0.0011 (2)	0.0009 (2)
C5C	0.0116 (3)	0.0072 (3)	0.0106 (3)	−0.0002 (2)	0.0004 (2)	0.0009 (2)
C6C	0.0113 (3)	0.0075 (3)	0.0116 (3)	−0.0007 (2)	0.0013 (2)	0.0003 (2)
S1	0.0115 (1)	0.0059 (1)	0.0067 (1)	0.0002 (1)	−0.0008 (1)	0.0003 (1)
O1	0.0199 (3)	0.0090 (2)	0.0111 (3)	−0.0048 (2)	−0.0008 (2)	0.0018 (2)
O2	0.0167 (2)	0.0094 (2)	0.0097 (3)	0.0013 (2)	0.0025 (2)	−0.0014 (2)
O3	0.0123 (2)	0.0111 (2)	0.0122 (3)	0.0025 (2)	−0.0013 (2)	−0.0024 (2)
O4	0.0174 (2)	0.0083 (2)	0.0076 (2)	0.0005 (2)	−0.0016 (2)	0.0019 (2)

### Geometric parameters (Å, °)

**Table:**

S1—O1	1.4706 (10)	N1C—C6C	1.3665 (12)
S1—O2	1.4706 (10)	N1C—C2C	1.3682 (12)
S1—O3	1.4895 (10)	N2C—C4C	1.3052 (12)
S1—O4	1.4905 (10)	N3C—C4C	1.3660 (12)
O5C—C2C	1.2281 (11)	N3C—C2C	1.3772 (13)
N1A—C2A	1.3649 (13)	N1C—H1C	0.864 (9)
N1A—C6A	1.3628 (12)	N2C—H4C	0.864 (8)
N2A—C6A	1.3184 (12)	N2C—H2C	0.864 (11)
N3A—C2A	1.3077 (12)	N3C—H3C	0.882 (8)
N3A—C4A	1.3568 (12)	C4A—C5A	1.3921 (12)
N7A—C8A	1.3245 (12)	C5A—C6A	1.4101 (12)
N7A—C5A	1.3787 (12)	C2A—H2A	0.9300
N9A—C4A	1.3616 (12)	C8A—H8A	0.9300
N9A—C8A	1.3634 (12)	C4C—C5C	1.4260 (12)
N1A—H1A	0.877 (10)	C5C—C6C	1.3548 (13)
N2A—H4A	0.858 (10)	C5C—H5C	0.9300
N2A—H3A	0.873 (9)	C6C—H6C	0.9300
N9A—H9A	0.872 (8)		
			
O1—S1—O4	107.88 (3)	N3A—C4A—C5A	126.85 (6)
O1—S1—O2	111.16 (3)	N3A—C4A—N9A	127.46 (6)
O1—S1—O3	109.72 (3)	N9A—C4A—C5A	105.70 (6)
O3—S1—O4	108.98 (3)	N7A—C5A—C4A	110.74 (6)
O2—S1—O3	109.20 (3)	N7A—C5A—C6A	131.12 (6)
O2—S1—O4	109.87 (3)	C4A—C5A—C6A	118.14 (6)
C2A—N1A—C6A	123.35 (6)	N1A—C6A—N2A	120.63 (6)
C2A—N3A—C4A	112.24 (6)	N2A—C6A—C5A	125.47 (6)
C5A—N7A—C8A	103.57 (6)	N1A—C6A—C5A	113.89 (6)
C4A—N9A—C8A	106.43 (6)	N7A—C8A—N9A	113.57 (7)
C2A—N1A—H1A	118.3 (7)	N3A—C2A—H2A	117.00
C6A—N1A—H1A	118.3 (7)	N1A—C2A—H2A	117.00
H3A—N2A—H4A	122.0 (11)	N7A—C8A—H8A	123.00
C6A—N2A—H3A	118.8 (8)	N9A—C8A—H8A	123.00
C6A—N2A—H4A	118.7 (7)	O5C—C2C—N3C	121.54 (6)
C4A—N9A—H9A	128.6 (8)	O5C—C2C—N1C	122.74 (7)
C8A—N9A—H9A	124.7 (8)	N1C—C2C—N3C	115.72 (6)
C2C—N1C—C6C	122.27 (7)	N2C—C4C—N3C	118.78 (6)
C2C—N3C—C4C	124.30 (6)	N2C—C4C—C5C	123.24 (7)
C6C—N1C—H1C	121.6 (8)	N3C—C4C—C5C	117.98 (7)
C2C—N1C—H1C	115.7 (8)	C4C—C5C—C6C	117.72 (7)
C4C—N2C—H2C	121.4 (7)	N1C—C6C—C5C	121.94 (6)
H2C—N2C—H4C	117.2 (10)	C4C—C5C—H5C	121.00
C4C—N2C—H4C	120.6 (7)	C6C—C5C—H5C	121.00
C2C—N3C—H3C	114.5 (7)	C5C—C6C—H6C	119.00
C4C—N3C—H3C	120.9 (7)	N1C—C6C—H6C	119.00
N1A—C2A—N3A	125.53 (7)		

### Hydrogen-bond geometry (Å, °)

**Table:**

*D*—H···*A*	*D*—H	H···*A*	*D*···*A*	*D*—H···*A*
N1A—H1A···O1^i^	0.877 (10)	2.535 (10)	3.1036 (19)	123.3 (8)
N1A—H1A···O4^i^	0.877 (10)	1.928 (11)	2.7833 (17)	164.5 (11)
N1C—H1C···O3	0.864 (9)	1.877 (10)	2.7350 (17)	172.0 (11)
N2C—H2C···O1^ii^	0.864 (11)	1.902 (11)	2.7596 (17)	171.8 (11)
N2A—H3A···N7A^iii^	0.873 (9)	2.081 (10)	2.9118 (18)	158.7 (12)
N3C—H3C···O4^iv^	0.882 (8)	1.835 (8)	2.7164 (17)	178.2 (11)
N2A—H4A···O5C^v^	0.858 (10)	2.102 (10)	2.8368 (18)	143.3 (9)
N2C—H4C···O2^iv^	0.864 (8)	1.901 (8)	2.7622 (17)	174.4 (11)
N9A—H9A···O3^vi^	0.872 (8)	1.870 (8)	2.7364 (17)	172.5 (12)
C2A—H2A···O1^i^	0.9300	2.3900	3.0553 (19)	128.00
C5C—H5C···O2^ii^	0.9300	2.4600	3.357 (2)	161.00
C6C—H6C···N3A^v^	0.9300	2.5300	3.447 (2)	170.00
C8A—H8A···O5C^iv^	0.9300	2.4200	3.245 (2)	148.00

Symmetry codes: (i) −*x*+1, −*y*, −*z*; (ii) −*x*+1, −*y*, −*z*+1; (iii) −*x*+2, −*y*, −*z*+1; (iv) *x*+1/2, −*y*+1/2, *z*+1/2; (v) −*x*+3/2, *y*−1/2, −*z*+1/2; (vi) −*x*+3/2, *y*+1/2, −*z*+1/2.
